# Five Criteria Predict Induction and Ablation of Supraventricular Tachycardia

**DOI:** 10.1111/jce.16496

**Published:** 2024-11-18

**Authors:** Thomas J. McGarry, T. Jared Bunch, Ravi Ranjan, Gregory J. Stoddard

**Affiliations:** ^1^ Division of Cardiology George E. Wahlen Veterans Affairs Medical Center Salt Lake City Utah USA; ^2^ Division of Cardiovascular Medicine University of Utah Health Sciences Center Salt Lake City Utah USA; ^3^ Division of Epidemiology University of Utah School of Medicine Salt Lake City Utah USA

**Keywords:** arrhythmia ablation, arrhythmia induction, electrophysiologic study, patient selection, prediction algorithm, supraventricular tachycardia

## Abstract

**Introduction:**

Current guidelines recommend electrophysiological study (EPS) and ablation for primary treatment of supraventricular tachycardia (SVT), but there is little information to guide patient selection for the procedure. The purpose of this study was to identify preoperative features that would predict whether patients with signs or symptoms of tachycardia were likely to have SVT induced and ablated at EPS.

**Methods:**

We performed a retrospective chart review of 1089 patients referred for EPS and ablation of SVT at 2 high volume centers. The population consisted of a derivation cohort of 810 patients and a validation cohort of 279 patients. We evaluated various clinical, EKG, and monitor features to determine which ones correlated with SVT induction or ablation.

**Results:**

Five preoperative findings predicted a high probability that SVT would be induced and ablated at EPS:

1. A characteristic EKG recording of SVT.
2. Termination of SVT with adenosine.
3. Termination of SVT or symptoms with vagal maneuvers.
4. An episode of SVT lasting ≥ 30 s on a monitor recording.
5. Pre‐excitation on the baseline EKG.

Patients exhibiting at least one of these features had a high probability of SVT induction and ablation, while those exhibiting none had a low probability (Induction, 76% vs. 19%, RR = 3.96 (2.76–5.69), *p* < .001; Ablation, 88% versus 26%, RR = 3.32 (2.48–4.46), *p* < .001). A point‐based score was derived and validated that can be used to estimate the probability of induction and ablation for individual patients.

**Conclusion:**

Simple criteria classify patients as having a high or low probability of SVT induction and ablation at EPS. They can be used as a guide for clinical decision making when considering invasive testing for patients with symptoms of tachycardia.

## Graphical abstract

Five criteria predict whether patients are likely or unlikely to have supraventricular tachycardia induced or ablated at an electrophysiologic study (EPS). The criteria can be used to select patients for invasive testing, to provide a rationale for avoiding EPS in high‐risk cases, and to manage patient and operator expectations.

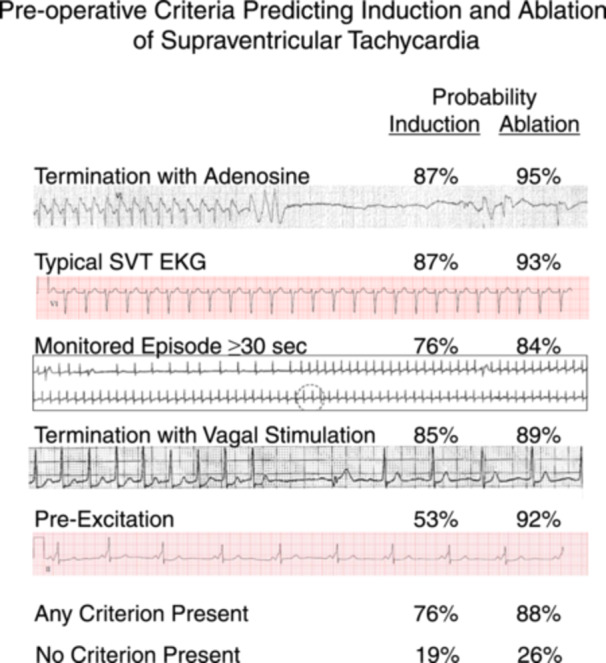

## 1 Introduction

Electrophysiological study (EPS) with ablation has supplanted medical management as the primary therapy for supraventricular tachycardia (SVT). Typical success rates range from 80% to 99% [1, 2, 3]. Unfortunately SVT cannot be induced in ~30% of patients who undergo EPS for evaluation of tachycardia [4, 5, 6, 7, 8]. In such cases, the operator cannot be certain whether the symptoms are from SVT or from another arrhythmia such as sinus tachycardia or atrial fibrillation (AF). Identifying an ablation target can be problematic or impossible. Empiric ablation of the slow pathway (SP) region carries the risk of damage to the atrioventricular node requiring a permanent pacemaker. Noninduction of SVT frustrates both the patient and the staff, unnecessarily consumes medical resources, and exposes patients to the inconvenience, discomfort, and risk of the procedure without the benefit of definitive therapy.

Current guidelines recommend EPS and ablation as first‐line therapy for patients with SVT but do not elaborate on which patients with signs and symptoms of tachycardia are most likely to benefit from the procedure [9, 10]. To guide patient selection, we sought to develop an algorithm for predicting whether SVT would be induced or ablated at EPS. In a large population of patients who underwent EPS for treatment of tachycardia, we evaluated a number of preoperative historical and clinical features to determine which ones predicted SVT induction and ablation.

## 2 Materials and Methods

### 2.1 Patients

The study population was 1300 consecutive patients who underwent EPS for evaluation of SVT at either the University of Utah Medical Center or the George E. Wahlen Veterans Administration Medical Center between January 1, 2010 and January 11, 2024. Cases were ascertained by reviewing scheduling records. Patients were excluded if they were referred for ablation of AF or atrial flutter (AFL); if comprehensive attempts at SVT induction were not performed; if SVT was incessant; if sustained ventricular tachycardia was induced; if the final diagnosis was dual atrioventricular nodal non‐reentrant tachycardia (DAVNNT) [11, 12]; or if the results of EPS were insufficiently documented. The study was approved by the Institutional Review Boards of the George E. Wahlen VA Medical Center and the University of Utah (IRB 00123357).

### 2.2 Data Collection and Definitions

Patient data were retrieved from the medical record, including referral documentation from outside institutions. For patients who underwent multiple EP studies, each follow‐up study was analyzed using only data that had accrued since the preceding study, unless no ablation had been performed or if ablation was known to have been unsuccessful. In these cases the study was analyzed using all available data. Intra‐operative electrograms were reviewed when necessary.

### 2.3 Presentation and Symptoms

Patients with an emergency room (ER) presentation came to the ER at least once on their own initiative complaining of symptoms of SVT (palpitations, chest discomfort, dyspnea, lightheadedness, or syncope). Those with a clinic presentation complained of symptoms but never had an ER presentation. All others had an incidental presentation, including patients with episodes of SVT noted at routine device interrogation and those with asymptomatic pre‐excitation. Presentations were considered incidental regardless of whether the patient reported symptoms retrospectively. Syncope was defined as a true loss of consciousness, regardless of whether it occurred in conjunction with SVT or how distantly it preceded EPS. SVT was considered have a sudden or non‐sudden onset as documented in the notes. Patients who reported that their symptoms were related to activity, drinking alcohol, or anxiety were classified as having a non‐sudden onset unless the notes explicitly stated otherwise. Patients who presented with syncope and concurrent SVT were excluded from the analysis of sudden onset.

### 2.4 EKG During SVT

EKGs recorded during episodes of SVT were classified as showing either (1) no visible P waves, (2) an rSr' pattern (VA interval < 80 msec, most commonly seen in leads V1, II, III, or aVF), (3) a short RP interval (RP < PR and VA > 80 msec), or (4) a long RP interval (RP > PR, Figure 2A–D). Mid‐RP tachycardias (RP = PR) were classified as short RP. SVT was considered irregular if the shortest RR interval was less than 85% of the longest RR interval [13].

### 2.5 Response to Adenosine or Vagal Maneuvers

Adenosine‐responsive patients had at least one documented episode when SVT terminated abruptly when they were given IV adenosine. Transient slowing of the tachycardia, delayed termination, and gradual termination were not considered positive responses. Adenosine nonresponsive patients had at least one documented episode when adenosine had failed and had never had a successful termination with adenosine. Patients whose tachycardia resolved with IV diltiazem, verapamil, metoprolol, procainamide, amiodarone, or electrical cardioversion were not considered adenosine‐responsive.

Vagal‐responsive and nonresponsive patients were defined in the same way as for adenosine. Abrupt termination of either tachycardia or symptoms was considered a positive response. Successful vagal maneuvers included valsalva, exhaling against resistance, deep breathing, coughing, carotid sinus massage, sudden exposure to cold stimuli, raising the legs, squatting, and placement of an IV catheter.

### 2.6 Monitor Recordings

Monitor recordings included those from ambulatory event monitors, implanted loop recorders, pacemakers, internal cardiac defibrillators, inpatient cardiac monitors, emergency medical system monitors, stress tests, and personal or wearable devices. P‐wave classifications and irregular rhythms were defined in the same way as for EKGs, using the monitored episode with the longest duration. Monitors were considered to show a correlation between SVT and symptoms if at least one episode was correlated regardless of its duration.

### 2.7 Pre‐Excitation

Patients were considered to exhibit pre‐excitation if the baseline EKG showed a clear delta wave or, if the delta wave was ambiguous, if the PR interval was < 120 msec and the QRS duration was > 120 msec. Patients whose EKGs were initially thought to show pre‐excitation but who had no evidence of an accessory pathway (AP) at EPS (*n* = 4) were re‐classified as not pre‐excited.

### 2.8 Electrophysiological Study

The details of the EPS were at the discretion of the individual operators, but generally included atrial and ventricular extrastimulus testing using isoproterenol when necessary at 0.5–40 µg/min titrated to increase the baseline heart rate by at least 20%. Dual antegrade atrioventricular node conduction pathways were considered to be present if during single atrial extrastimulus testing a decrement of 10 msec in the S_1_–S_2_ interval led to a ≥ 50 msec increase in the A_2_–H_2_ or H_1_–H_2_ interval. An AP was considered to be present if there was manifest pre‐excitation, if the retrograde atrial activation sequence during RV pacing was eccentric, if para‐Hisian pacing was consistent with an AP [14], or if atrioventricular re‐entrant tachycardia (AVRT) was induced.

### 2.9 Inducible Versus Noninducible Patients

Patients were classified as having SVT induced at EPS if either of these two criteria were met:

1) SVT lasted long enough that at least one diagnostic pacing maneuver could be performed. Patients were still considered inducible if SVT terminated during diagnostic pacing, if diagnostic pacing failed to entrain the tachycardia, or if SVT had to be terminated by rapid pacing or by cardioversion because of hemodynamic instability. Diagnostic pacing maneuvers did not include instances when a preprogrammed pacing train inadvertently impinged on a run of SVT induced by a previous train, even if SVT terminated during pacing.

2) SVT lasted at least 30 s.

All other patients were considered noninducible, including patients who had evidence of dual antegrade atrioventricular nodal pathways and one or more ECHO beats with atrial extrastimulus testing; patients with frequent premature atrial contractions or repeated runs of focal atrial tachycardia (FAT) each lasting less than 30 s; and patients who had only AF or AFL induced.

### 2.10 Empiric SP Ablation

An empiric ablation in a noninducible patient was considered appropriate if both of these criteria were met [15]:

1. A tracing consistent with atrioventricular nodal re‐entrant tachycardia (AVNRT, regular and non‐long RP tachycardia regardless of duration) had been previously recorded on a 12‐lead EKG or a monitor.
2. At EPS there was evidence of dual antegrade AV nodal conduction pathways, manifest as either
  a an A_2_–H_2_ or H_1_–H_2_ jump on atrial extrastimulus testing as described above, or
  b one or more atrial ECHO beats with a VA interval ≤ 70 msec.

All other empiric SP ablations were considered inappropriate.

### 2.11 Statistical Methods

We modeled the data using Poisson regression, with a binary outcome and robust standard errors. This model allowed us to compute risk ratios (RRs) directly, which are more intuitive than odds ratio from logistic regression. We derived a risk score from our multivariable Poisson regression, with score points based on the size of the regression coefficients and the RRs [16, 17]. For the patient characteristics (Supporting Information S1: Table S1), comparisons of unordered categorical variables were made using a chi‐square test or Fisher's exact test, as appropriate; a Wilcoxon‐Mann‐Whitney test for ordered categorical variables; and a two‐sample *t*‐test for continuous variables.

## 3 Results

### 3.1 Predicting SVT Induction

We evaluated a number of variables which, in our judgment, were likely to predict SVT induction and ablation. These included features found on the 12 lead EKG, the results of ambulatory or other cardiac monitors, the response to adenosine or vagal maneuvers, and clinical features such as the mode of presentation and the patient's description of their symptoms.

**Figure 1:**
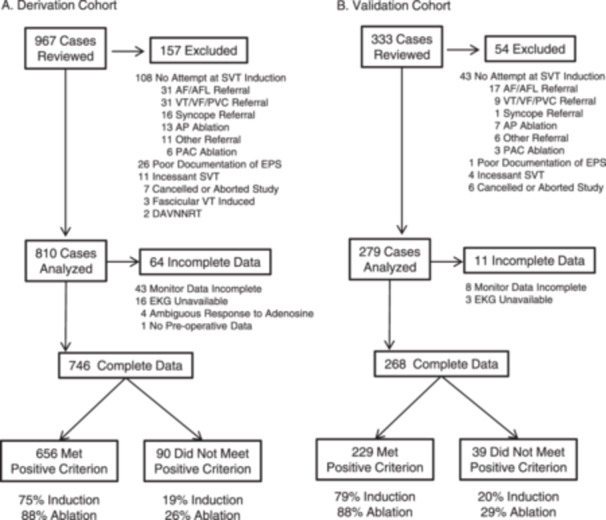
Patient cohorts and overall results. (A) Derivation cohort. (B) Validation cohort. AF, atrial fibrillation; AFL, atrial flutter; AP, accessory pathway; DAVNNRT, dual AV nodal nonreentrant tachycardia; PAC, premature atrial contraction; PVC, premature ventricular contraction; SVT, supraventricular tachycardia; VT, ventricular tachycardia; VF, ventricular fibrillation.

The total study population consisted of a derivation cohort (cases from 1/1/10 to 9/13/21) and a validation cohort (cases from 9/14/21 to 1/11/24). For the derivation cohort, the records of 967 patients who were referred for EPS for SVT were screened for inclusion in the study. Of these, 157 were excluded for the reasons outlined in Figure 1A. Of the remaining 810 patients, 547 (68%) had SVT induced at EPS and 263 (32%) did not. Baseline characteristics were similar between the inducible and noninducible groups (Supporting Information S1: Table S1), except the noninducible patients were slightly younger (average age 48.4 vs. 53.0 years).

About half of the SVT inductions required isoproterenol (average 3.2, median 2 mcg/min). Most cases of non‐induction (87%) employed isoproterenol at a higher dose (average 5.7, median 4 mcg/min). Half of the non‐inducible patients who were not treated with isoproterenol (16/32) showed manifest pre‐excitation.

Among the inducible patients, 94% met the criterion that tachycardia lasted long enough that a diagnostic pacing maneuver could be performed and 6% met the criterion that tachycardia lasted at least 30 s. Using our definition of SVT induction, 85%–95% of patients who underwent an appropriate ablation had SVT induced (Supporting Information S1: Table S2).

### 3.2 The 12 Lead EKG

**Figure 2:**
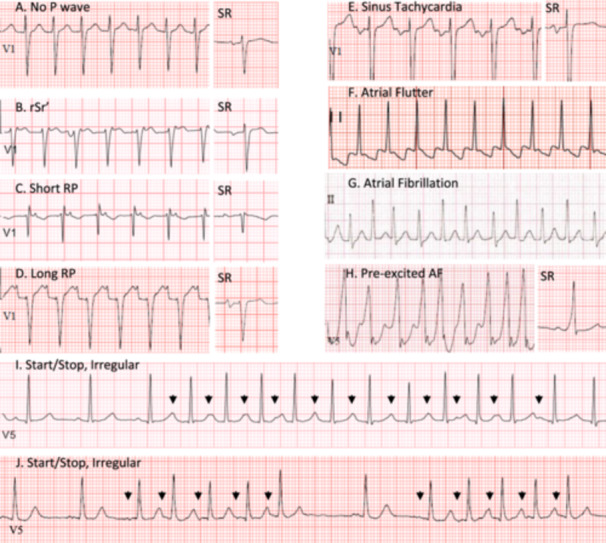
Typical and non‐typical SVT EKG patterns. (A–D) Typical SVT EKGs. (E–J) non‐typical SVT EKGs. Sinus rhythm (SR) complexes from the same lead are shown for comparison. Arrowheads indicate P waves.

**Figure 3:**
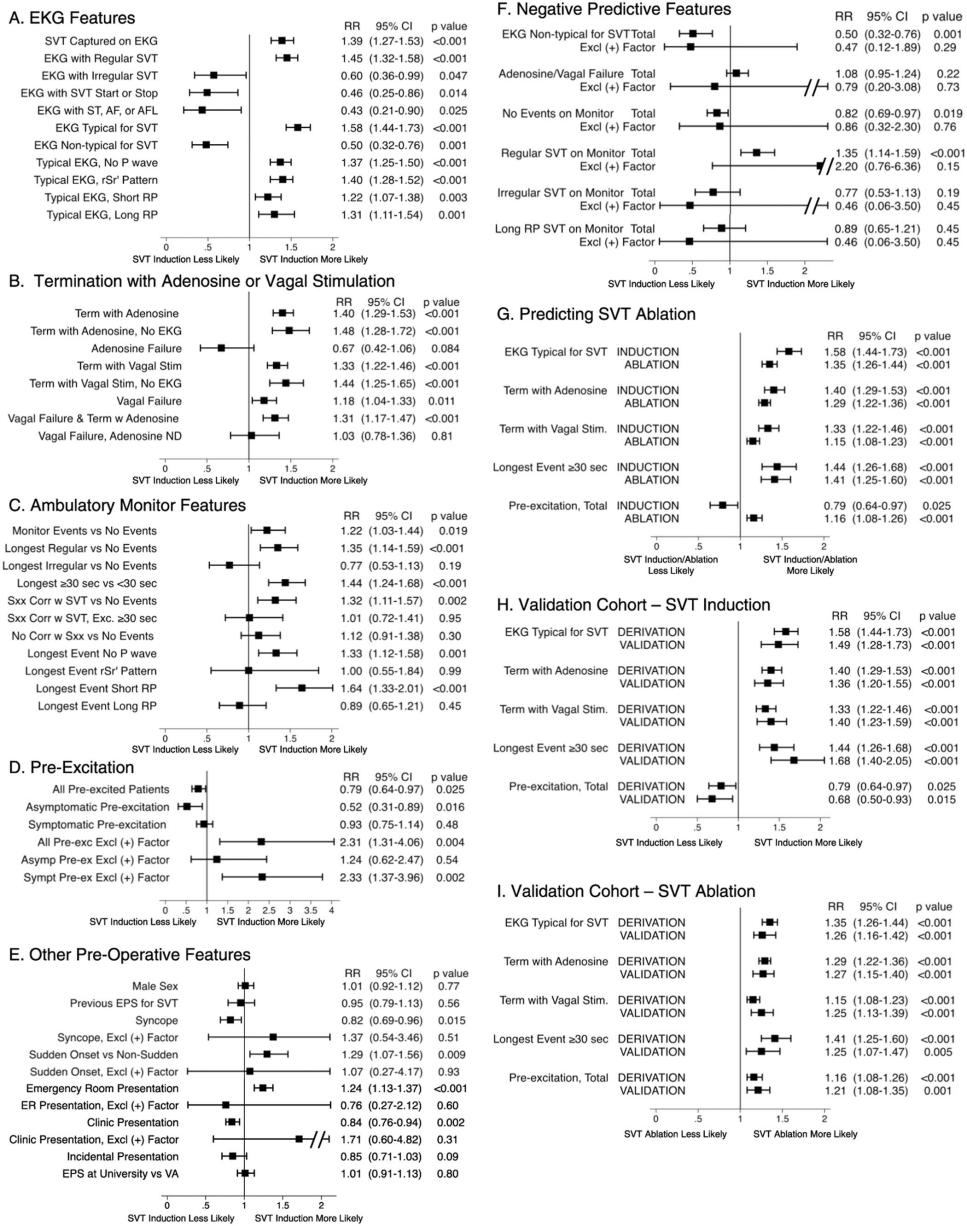
Preoperative factors predicting SVT induction and ablation. Patients who exhibited each feature were compared to all patients who did not, including those with no data, except as noted below. The RR values, 95% confidence limits, and *p* values were calculated using Poisson regression. Asympt, Asymptomatic; CI, Confidence Interval, Excl, Excluding; RR, Risk Ratio; Sxx, Symptoms; Sympt, Symptomatic; Term, Termination. (A) An EKG showing regular and uninterrupted SVT predicts SVT induction. (B) Termination with adenosine or vagal stimulation predicts SVT induction. ND, No Data. (C) Regular SVT episodes on monitoring predict SVT induction. Patients exhibiting each feature were compared to patients who had no episodes on monitoring. (D) Pre‐excitation predicts SVT induction after other variables are excluded. (+) factor indicates typical SVT EKG, termination with adenosine or vagal stimulation, or monitored event ≥ 30 s. Note change in scale. (E) Preoperative factors that do not independently predict SVT induction. (+) factor indicates typical SVT EKG, termination with adenosine or vagal stimulation, monitored event ≥ 30 s, or pre‐excitation. (F) Negative predictive factors are non‐informative. (G) Features that predict SVT induction also predict SVT ablation. (H–I) The same features predict SVT induction and ablation in both the derivation and validation cohorts.

Patients who had SVT documented on a preoperative 12 lead EKG were more likely to have SVT induced at EPS than patients who did not (Figure 3A, Table 1A). Most SVT EKGs (87%) showed a regular tachycardia that persisted the entire duration of the recording, and most patients with this typical EKG pattern (87%) had SVT induced. Among patients with typical SVT EKGs, those exhibiting all types of P wave patterns (no P wave, rSr', short RP, or long RP, Figure 2A–D) were highly likely to have SVT induced (80%–90%).

**Table 1:** Factors predicting SVT induction, derivation cohort.

Variable	Variable present		Variable absent		RR	*p* Value
	Inducible/Total	Inducible %	Inducible/Total	Inducible %		
**A. EKG features**						
SVT captured on EKG	296/372	80	249/436	57	1.39	< 0.001
EKG with regular SVT	251/300	84	294/508	58	1.45	< 0.001
EKG with irregular SVT	9/22	41	536/786	68	0.60	0.047
EKG showing start or stop	7/22	32	538/786	68	0.46	0.014
EKG c/w sinus tachycardia, AF, or AFL	5/17	29	540/791	68	0.43	0.025
Typical SVT EKG	246/283	87	263/477	55	1.58	< 0.001
Non‐typical SVT EKG	14/41	34	495/719	69	0.50	0.001
Typical EKG, no P wave	90/102	88	455/706	64	1.37	< 0.001
Typical EKG, rSr' pattern	88/98	90	457/710	64	1.40	< 0.001
Typical EKG, short RP	54/67	81	491/741	66	1.22	0.003
Typical EKG, long RP	21/24	88	524/784	67	1.31	0.002
**B. Termination with Adenosine or Vagal Maneuvers**						
Termination with adenosine	198/233	85	347/573	61	1.40	< 0.001
Termination with adenosine without SVT EKG	49/61	80	250/462	54	1.48	< 0.001
Failure to terminate with adenosine	10/22	45	535/784	68	0.67	0.084
Termination with vagal stimulus	181/219	83	366/591	62	1.33	< 0.001
Termination with vagal stimulus without SVT EKG	91/122	75	210/405	52	1.44	< 0.001
Failure to terminate with vagal stimulus	67/86	78	480/724	66	1.18	0.011
Vagal failure with termination by adenosine	46/53	87	499/753	66	1.31	< 0.001
Vagal failure without termination by adenosine	20/32	63	327/541	60	1.03	0.81
**C. Cardiac monitor features**						
Monitor events versus no events	216/320	68	78/141	55	1.22	0.019
Longest event regular versus no events	169/227	74	78/141	55	1.35	< 0.001
Longest event irregular versus no events	18/42	43	78/141	55	0.77	0.19
Longest event ≥ 30 s versus < 30 s	195/268	73	104/206	50	1.44	< 0.001
Longest event ≥ 30 s without SVT EKG or adenosine/vagal termination	99/157	63	28/107	26	2.41	< 0.001
Symptoms correlated with SVT versus no events	140/192	73	78/141	55	1.32	0.002
Symptoms correlated with SVT versus no events, excluding longest event ≥ 30 s	19/34	56	78/141	55	1.01	0.95
Symptoms not correlated with SVT versus no events	65/105	62	78/141	55	1.12	0.30
Longest event No P wave versus no events	135/184	73	78/141	55	1.33	0.001
Longest event rSr' pattern versus no events	5/9	56	78/141	55	1.00	0.99
Longest event short RP pattern versus no events	19/21	90	78/141	55	1.64	< 0.001
Longest event long RP pattern versus no events	27/55	49	78/141	55	0.89	0.45
**D. Pre‐excitation**						
All pre‐excited patients	43/79	54	492/713	69	0.79	0.025
Asymptomatic pre‐excitation	9/25	36	526/767	69	0.52	0.016
Symptomatic pre‐excitation	34/54	63	501/738	68	0.93	0.48
All pre‐excited patients Excl. SVT EKG, adenosine/vagal termination, or event ≥ 30 s	21/51	41	15/84	18	2.31	0.004
Asymptomatic pre‐excitation, excluding those with (+) feature	7/22	32	29/113	26	1.24	0.54
Symptomatic pre‐excitation, excluding those with (+) feature	14/29	48	22/106	21	2.33	0.002
**E. Clinical features**						
Age ≥ 50	322/454	71	225/356	63	1.12	0.02
Age ≥ 50 excluding (+) factor	10/46	22	28/95	29	0.74	0.35
Male sex versus female sex	291/428	68	256/382	67	1.01	0.77
Previous EPS for SVT	47/73	64	500/737	68	0.95	0.56
History of syncope	68/120	57	479/690	69	0.82	0.015
History of syncope excluding (+) factor	5/21	24	12/69	17	1.37	0.51
Sudden onset versus not sudden	184/255	72	56/100	56	1.29	0.009
Sudden onset versus not sudden excluding (+) factor	4/20	20	3/16	19	1.07	0.93
ER presentation	313/420	75	234/390	60	1.24	< 0.001
ER presentation excluding (+) factor	4/26	15	13/64	20	0.76	0.60
Clinic presentation	180/298	60	367/512	72	0.84	0.002
Clinic presentation excluding (+) factor	13/59	22	4/31	13	1.71	0.31
Incidental presentation	49/84	58	498/726	69	0.85	0.09
EPS at university versus VA	408/602	68	139/208	67	1.01	0.80

In contrast, patients whose SVT EKG did not fit this typical pattern had a lower likelihood of SVT induction (Figure 2E–J). This group included patients whose tachycardia was irregular, patients whose tachycardia initiated or terminated during the recording, and patients whose tachycardia showed an EKG pattern consistent with sinus tachycardia, AF, or AFL. About 2/3 of the irregular tachycardias either started or stopped during the 10‐second recording period; conversely, about 2/3 of the tachycardias that started or stopped during recording were irregular. About half the patients whose tachycardia started or stopped during the recording had another EKG taken during the same clinical encounter that showed a continuous recording. Overall, only 34% of patients with a non‐typical EKG pattern were inducible.

### 3.3 Response to Adenosine or Vagal Maneuvers

Patients whose tachycardia terminated with adenosine were more likely to have SVT induced at EPS than other patients (Figure 3B, Table 1B). Patients whose tachycardia did not respond to adenosine showed a nonsignificant trend towards being less likely to have SVT induced.

Patients whose tachycardia terminated with a vagal maneuver were also more likely to have SVT induced. Patients who did not respond to vagal maneuvers, however, were still more likely to have SVT induced than the rest of the population. Notably, almost all vagal non‐responders who were subsequently given adenosine had their tachycardia terminate (53 of 58, 91%). This suggests that in many cases vagal stimulation was merely insufficient to terminate the arrhythmia. When patients whose tachycardia terminated with adenosine were excluded, vagal non‐responders were no more likely to have SVT induced at EPS than the others.

When patients with typical SVT EKGs were excluded from the analysis, adenosine‐ or vagal‐responsive patients were still more likely to have SVT induced than the others (Figure 3B), indicating that these are independent predictors of SVT induction.

### 3.4 Cardiac Monitors

Unlike EKGs, which have a standardized format, various types of monitors differ in the duration of the recording, in the number and position of the leads, and in the type of information reported about each event. Because of this variability, it can be problematic to directly compare results obtained from different types of monitors. In general we did not have complete information on every event, so we focused on the characteristics of the SVT episode with the longest duration, reasoning that this most likely reflected the patient's clinical tachycardia. The comparison (control) group was those whose monitors did not record any SVT episodes.

Patients who had episodes of SVT recorded on ambulatory monitoring were more likely to have SVT induced at EPS than patients who had no episodes (Figure 3C, Table 1C). As with the EKGs, for most patients (85%) the longest episode was a regular tachycardia. Patients whose longest episode was irregular were no more likely to have SVT induced than those who had no episodes at all; in fact there was as trend towards SVT induction being less likely. Those whose longest episode showed no visible P waves or a short RP tachycardia were more likely to have SVT induced, but those with a long RP tachycardia were not.

**Figure 4:**
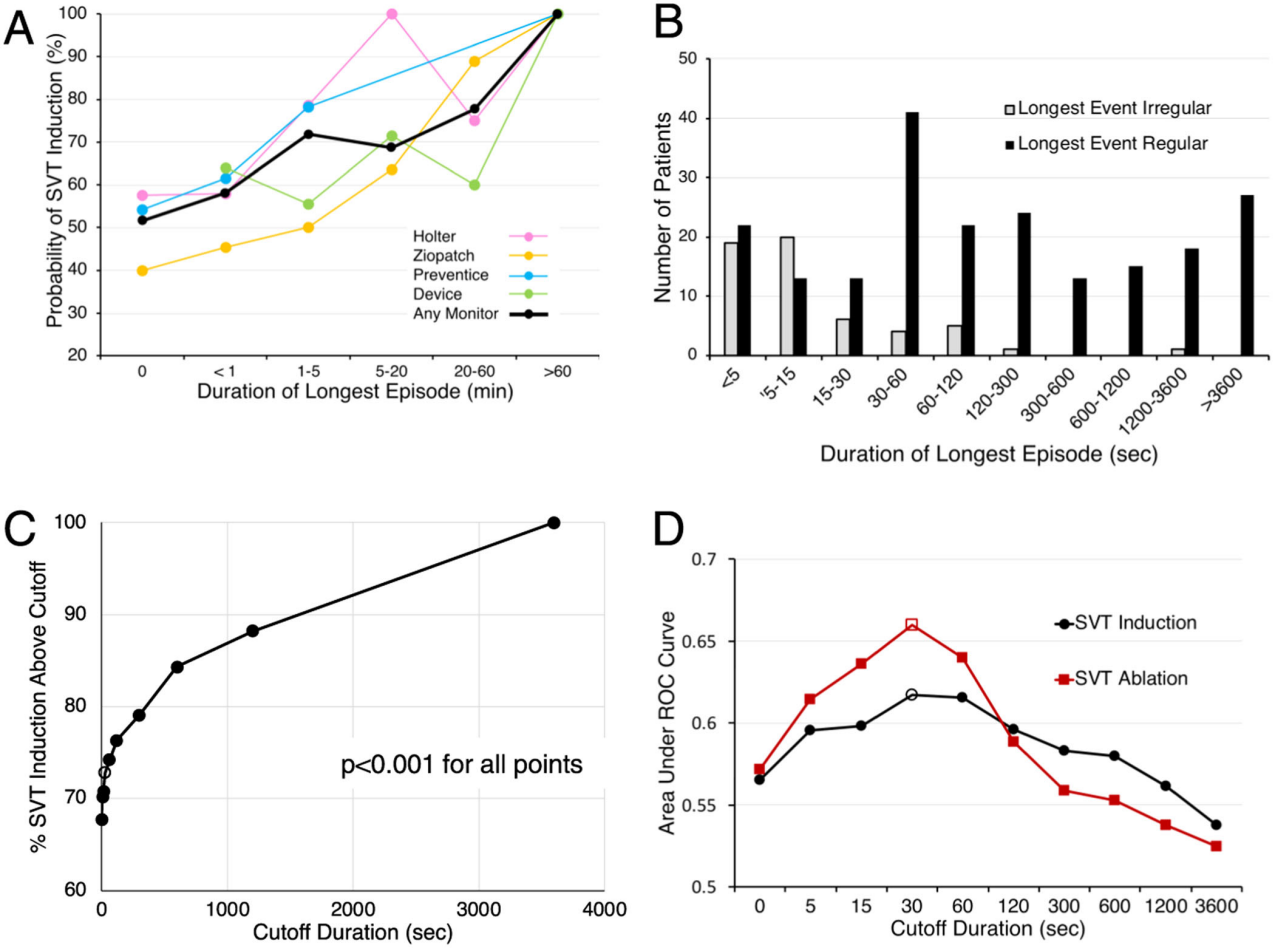
The duration of the longest episode on monitoring predicts SVT induction. (A) Patients with longer duration episodes are more likely to have SVT induced at EPS. Pastel lines show the results from different types of monitors. The solid black line shows the pooled results from all monitors. (B) Regular SVT events (black) tend to last longer than irregular events (gray). The duration of each patient's longest event is plotted. (C) The probability of SVT induction above each cutoff duration is plotted. The data points represent cutoff durations of 0, 5, 15, 30, 60, 120, 300, 600, 1200, and 3600 s. The open circle represents a cutoff of ≥ 30 s. When comparing the probability of SVT induction above and below the cutoff value, the *p* value was < 0.001 for all data points. (D) A cutoff duration of 30 s best distinguishes patients who will have SVT induced and ablated from those who will not. The vertical axis shows the area under the ROC curve for each cutoff duration. Black circles, SVT induction; red squares, SVT ablation. The open symbols represent a cutoff of ≥ 30 s.

The monitor parameter that best predicted SVT induction was the duration of the longest recorded episode. The longer this episode, the more likely that SVT would be induced (Figure 4A). We tested a number of cutoff duration values to see which would best distinguish between inducible and non‐inducible patients. For this analysis we also included results obtained from implantable devices, personal or wearable devices, cardiac monitors, and stress tests. The probability of inducing tachycardia above the cutoff duration rose rapidly at low values then plateaued (Figure 4C). The cutoff duration that gave the largest area under the receiver operator characteristic curve (AUC) was 30 s (AUC = 0.62, Figure 4D, open circle). When patients who had a characteristic SVT EKG or whose SVT terminated with adenosine or vagal maneuvers were excluded, the optimal cutoff remained the same, the AUC increased to 0.68, and the likelihood of inducing tachycardia relative to the remaining population increased (Table 1C, RR = 2.41 vs. 1.44). This indicates that a monitored episode lasting ≥ 30 s predicts SVT induction independently of the other factors.

**Figure 5:**
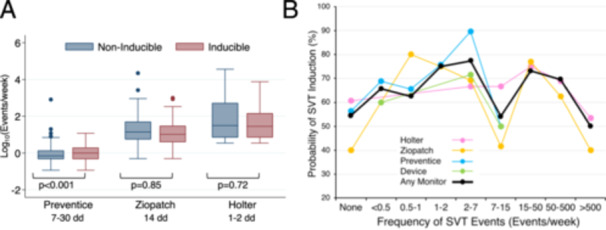
The frequency of SVT episodes on monitoring does not predict SVT Induction. (A) The frequency of SVT events on monitoring does not differ between patients who have SVT induced (pink) and those who do not (blue). Note that the frequency of SVT events varies widely between different types of monitors (the vertical axis is a logarithmic scale). *p* values were calculated using Student's *t*‐test assuming unequal variances, excluding outliers that were > 3× the interquartile range from the median. (B) The probability of SVT induction does not increase at higher event rates. Pastel lines show the results from different types of monitors. The solid black line shows the pooled results from all monitors. The lower frequency range is dominated by Preventice monitors, the middle range by Ziopatch monitors, and the upper range by Holter monitors.

No other monitor feature independently predicted SVT induction (Figure 3C). The frequency of SVT episodes varied widely among different types of monitors, undoubtedly reflecting different criteria as to what constitutes a reportable event (Figure 5A). The frequency of episodes was not helpful in predicting whether SVT would be induced even among monitors from the same manufacturer (Figure 5A,B). One brand of monitor (Preventice) showed a small difference in event frequency between non‐inducible and inducible patients, but this difference disappeared once patients with episodes lasting ≥ 30 s were excluded (not shown). Patients who reported symptoms that correlated with episodes of SVT were significantly more likely to have SVT induced than patients without episodes or patients without a correlation, but these differences also disappeared when patients with episodes ≥ 30 s were excluded.

### 3.5 Pre‐Excitation

Only about half (54%) of the pre‐excited patients had SVT induced at EPS, a rate slightly but significantly lower than that of non‐pre‐excited patients (Figure 3D, Table 1D). This seemingly counterintuitive result is driven by patients who had incidentally discovered asymptomatic pre‐excitation; those with symptomatic pre‐excitation were equally likely to have SVT induced as non‐pre‐excited patients.

The overall probability of SVT induction in the derivation cohort was quite high (68%), which could mask any propensity for pre‐excited patients to have SVT induced. To see if this was the case, we repeated the analysis after excluding patients who exhibited any of the positive predictive criteria described previously (typical SVT EKG, monitored episode of SVT lasting ≥ 30 s, or termination with adenosine or vagal maneuvers). We also excluded patients in whom the preoperative evidence was indeterminate (Figure 1A). Indeterminate cases included, for example, those where an EKG was taken during SVT but it was not available for review or those where episodes of SVT were reported on monitoring but it was not known whether any lasted ≥ 30 s. After excluding positive and indeterminate cases, the patients with pre‐excitation were about twice as likely to have SVT induced at EPS as those without (Figure 3D).

### 3.6 Other Clinical Factors

We found no other factors that independently predicted SVT induction. Neither the patient's age, the patient's sex, the heart rate during tachycardia, the peak serum troponin‐I (TnI) level after an episode, a history of a previous EPS for SVT, the site of EPS (VA vs. University), nor the operator affected the probability of SVT induction (Figure 3E, Table 1E, and not shown). Patients with a history of sudden onset tachycardia or an ER presentation were more likely to have SVT induced than the others while patients with syncope or a clinic presentation were less likely, but none of these features independently predicted SVT induction when patients with one of the positive predictive factors and indeterminate patients were excluded.

### 3.7 Factors Predicting Non‐Induction of SVT

To search for additional positive or negative predictive factors, we repeated the analysis on the total population after excluding patients who exhibited any of the 5 positive predictive criteria described above and indeterminate cases. We could not find any additional predictive factors (Figure 3F, Supporting Information S1: Table S3), perhaps because there were only 89 patients in this group. There were nonsignificant trends towards SVT induction being less likely in patients with a non‐typical SVT EKG, an irregular tachycardia on monitoring, or a long RP tachycardia on monitoring. There was also a nonsignificant trend towards SVT induction being more likely in patients with a regular SVT episode recorded on a monitor.

### 3.8 Predicting SVT Ablation

More patients in the derivation cohort underwent an ablation than had SVT induced (80% vs. 67%). To see if our criteria would also predict SVT ablation, we first adjusted the data by censoring 19 inappropriate empiric SP ablations and adding 14 empiric SP ablations that would have been appropriate but were not performed (see Methods).

The features that predicted SVT induction also predicted whether an ablation would be performed (Figure 3G, Supporting Information S1: Table S4). Patients with pre‐excitation, though overall less likely to have SVT induced, were more likely to undergo ablation than patients without pre‐excitation. For cardiac monitors, the optimal cutoff episode duration that distinguished patients who underwent ablation from those who did not was 30 s (AUC = 0.66, Figure 5D). Criteria that were non‐predictive for SVT induction were also non‐predictive for SVT ablation.

### 3.9 Prediction Score

**Figure 6:**
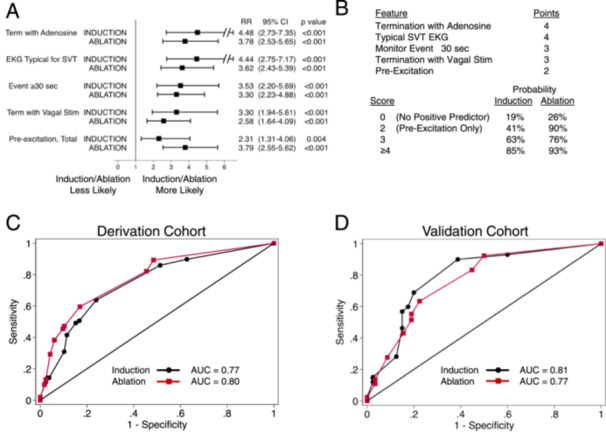
Scoring system for estimating the probability of SVT induction and ablation. (A) Each preoperative feature independently predicts SVT induction and ablation. The RR and *p* values for each feature were calculated after excluding patients who exhibited any of the other features. (B) Algorithm for estimating the probability of SVT induction or ablation. Points in the prediction score were calculated by dividing each regression coefficient (Supporting Information S1: Table S4) by the smallest coefficient, multiplying by 2 to be accurate within 0.5, and rounding to the nearest integer. (C) ROC curves showing the performance of the prediction score in predicting SVT induction (black circles) or ablation (red squares) in the derivation cohort. (D) Same as (C), validation cohort.

To develop a scoring system, we calculated the RR values for each individual feature independent of the others by excluding patients who had any of the other features. Each feature independently predicted SVT induction and ablation with RR values ranging from 2.0 to 5.0 (Figure 6A and Supporting Information S1: Table S5). We assigned point values for each feature based on the RR values for SVT induction (Figure 6B). Using the regression coefficients resulted in the same point values. The highest point values were for a history of termination with adenosine and a typical SVT EKG, and the lowest was for pre‐excitation. The total score was highly correlated with the probability of SVT induction (AUC = 0.77) or ablation (AUC = 0.80, Figure 6C). For patients who did not exhibit any positive predictive factors (Score = 0), the probabilities of inducing and ablating SVT were only 19% and 26% respectively (Figure 6B). For patients whose only positive predictive factor was pre‐excitation (Score = 2), the probabilities rose to 41% and 90%. For any Score ≥ 3, the probability of inducing and ablating SVT at EPS was > 60%.

### 3.10 Validation Cohort

To confirm our findings, we analyzed a validation cohort of 279 new patients using the same methodology (Figure 1B). Although the number of patients was smaller, we were able to demonstrate the same statistically significant correlations between all of the factors we analyzed and the likelihood of SVT induction with the exception of pre‐excitation (Figure 3H, Supporting Information S1: Table S6). Even after excluding patients with another positive predictive factor, pre‐excited patients were no more likely to have SVT induced than the non‐pre‐excited patients in the validation cohort (Figure 3H), though they were still more likely in the total population (derivation + validation cohorts, not shown). All features that predicted a high likelihood of SVT ablation in the derivation cohort also predicted high likelihood of SVT ablation in the validation cohort (Figure 3I, Supporting Information S1: Table S7). The total point score was also highly correlated with SVT induction and ablation in the validation cohort (AUC of 0.81 and 0.77 respectively, Figure 6D).

## 4 Discussion

EPS and ablation are considered primary therapy for SVT but there are no guidelines to identify the patients who are most likely to benefit from the procedure. In published studies, SVT cannot be induced in ~30% of patients referred for EPS. Non‐induction of SVT can make definitive therapy through ablation problematic or unfeasible.

In this study we identified five preoperative features that strongly predict that a patient is likely to have SVT induced and ablated at EPS:

1. A characteristic EKG recording of SVT (regular tachycardia lasting the entire duration of the tracing and not consistent with sinus tachycardia, AF, or AFL).

2. Termination of SVT with adenosine.

3. Termination of SVT or symptoms with vagal maneuvers.

4. A monitored episode of SVT lasting ≥ 30 s.

5. Pre‐excitation on the baseline EKG.

Considered in aggregate, patients exhibiting at least one of these 5 features had a 76% probability of SVT induction and an 88% chance of SVT ablation, while those who did not exhibit any had only a 19% chance of induction and a 26% chance of ablation (Figure 1A).

Table 2 lists the sensitivity, specificity, positive predictive value (PPV), and negative predictive value (NPV) for each of the individual features calculated over the total population. Termination with adenosine and a typical SVT EKG had the highest sensitivity and PPV (85%–95%). Termination with vagal stimulation had a high PPV (85%) but a low NPV (21%), reflecting the fact that vagal stimulation is often insufficient to terminate SVT. A monitored event lasting ≥ 30 s had a moderate PPV and NPV (76% and 49%), reflecting the sporadic nature of SVT events. Pre‐excitation was highly predictive of an ablation being performed, but only modestly predictive of SVT induction.

**Table 2:** Statistical parameters.

Typical SVT EKG	SVT induction				SVT ablation			
	Induced		Not induced		Ablated		Not ablated	
Typical SVT EKG	339		50		362		27	
Non‐typical SVT EKG	19		34		27		26	
	**Sens**	**Spec**	**PPV**	**NPV**	**Sens**	**Spec**	**PPV**	**NPV**
	95%	40%	87%	64%	93%	49%	93%	49%
**Termination with adenosine**	**Induced**		**Not induced**		**Ablated**		**Not ablated**	
Termination with adenosine	266		44		293		17	
Non‐term with adenosine	19		15		22		12	
	**Sens**	**Spec**	**PPV**	**NPV**	**Sens**	**Spec**	**PPV**	**NPV**
	93%	25%	86%	44%	93%	41%	95%	35%
**Termination with vagal stim**	**Induced**		**Not induced**		**Ablated**		**Not ablated**	
Termination with vagal stim	246		45		260		31	
Non‐term with vagal stim	93		24		102		15	
	**Sens**	**Spec**	**PPV**	**NPV**	**Sens**	**Spec**	**PPV**	**NPV**
	73%	35%	85%	21%	72%	33%	89%	13%
**Longest monitor event** ≥ **30**	**Induced**		**Not induced**		**Ablated**		**Not ablated**	
Longest Event ≥ 30 s	282		89		313		58	
Longest Event < 30 s	159		155		196		118	
	**Sens**	**Spec**	**PPV**	**NPV**	**Sens**	**Spec**	**PPV**	**NPV**
	64%	64%	76%	49%	61%	67%	84%	38%
**Pre‐excitation**	**Induced**		**Not induced**		**Ablated**		**Not ablated**	
Pre‐excitation	63		56		109		10	
No pre‐excitation	664		278		734		208	
	**Sens**	**Spec**	**PPV**	**NPV**	**Sens**	**Spec**	**PPV**	**NPV**
	9%	83%	53%	30%	13%	95%	92%	22%
**Aggregate of All five features**	**Induced**		**Not induced**		**Ablated**		**Not ablated**	
≥ 1 predictive factor present	676		213		780		109	
No predictive factor present	24		101		33		92	
	**Sens**	**Spec**	**PPV**	**NPV**	**Sens**	**Spec**	**PPV**	**NPV**
	97%	32%	76%	81%	96%	46%	88%	74%

We generated a point‐based score system for estimating the preoperative probability that SVT will be induced or ablated (Figure 6B). The score reflects the relative predictive strength of each feature. Patients with any score ≥ 3 (i.e., any individual feature except pre‐excitation) have a probability of SVT induction and ablation of at least 60%. Other scoring systems based on patient questionnaires agree that those who report documentation of tachycardia, termination with medications or vagal maneuvers, or longer symptomatic episodes are more likely to have SVT induced [18, 19].

In many respects our findings corroborate what would be expected from clinical intuition. One common thread is that longer duration tachycardia episodes predict successful SVT induction and ablation. We found that the cutoff duration of a monitored episode that best distinguishes inducible from noninducible patients is 30 s (Figure 4D). Some of other positively predictive features also reflect longer duration episodes. For example, to have a 12 lead EKG taken during SVT or to be given adenosine, the patient's tachycardia would have to last long enough for them to seek medical attention and undergo at least a rudimentary evaluation.

Termination of tachycardia by adenosine strongly suggests that the arrhythmia is a form of SVT that uses the AV node as part of the circuit, such as AVNRT or AVRT [20, 21]. Adenosine also suppresses triggered activity and can terminate the majority of FATs, many of which have a triggered mechanism [22, 23, 24]. Adenosine causes only transient slowing of other common narrow complex tachycardias such as sinus tachycardia, AF, or AFL. Vagus nerve stimulation also slows AV conduction and suppresses triggered activity, but not to the same extent as adenosine [25].

In other respects our findings run counter to clinical intuition. The frequency of SVT events on monitoring is poorly correlated with induction, probably because the number of sustained episodes is overwhelmed by a high background of short bursts of atrial tachycardia. Patients who report more frequent symptoms are more likely to have SVT induced [19], so if short bursts were screened out such a correlation might exist. Sudden onset of symptoms does not by itself predict successful induction or ablation of SVT, nor does a history of syncope. The mode of presentation (ER, clinic, or incidental) makes no difference. Failure of an arrhythmia to terminate with adenosine or vagal stimulation carries less information than termination. In such cases the arrhythmia mechanism may not have been of a type that would respond, or the effect of the intervention may have been insufficient to cause termination.

Perhaps most importantly, our findings identify a group of patients in whom induction or ablation of SVT is unlikely and in whom EPS could be avoided. These patients characteristically have shorter recorded episodes of SVT. Patients with shorter bursts of SVT would be more likely to have the initiation or termination captured on a 12 lead EKG, which we find suggests non‐induction. A second characteristic of non‐inducible patients is an irregular tachycardia. Irregularity was associated with short duration episodes; about 2/3 of the irregular tachycardias recorded on an EKG started or stopped while the EKG was being recorded and vice‐versa. Additionally, irregular episodes of tachycardia captured on monitoring tended to be shorter than regular episodes (Figure 4B). Irregularity suggests that the tachycardia mechanism is non‐reentrant FAT [13], either because of variable AV block or because the tachycardia focus itself fires irregularly (Figure 2 I‐J). Patients with an irregular SVT noted pre‐operatively were more likely to have FAT diagnosed at EPS than the total population (not shown). Nevertheless, ~85% of preoperative recordings from patients diagnosed with FAT showed a regular SVT.

Only ~50% of pre‐excited patients had SVT induced at EPS, as reported previously [5]. Because an AP can be targeted for ablation without inducing tachycardia, induction attempts may have been less exhaustive in this group. We found, for example, that among the non‐inducible patients only 63% of those with pre‐excitation had isoproterenol used during induction attempts compared to 92% of those without pre‐excitation. Our data may underestimate the probability of inducing tachycardia in pre‐excited patients. Ablating an AP removes a small risk of sudden cardiac death [26], so referring pre‐excited patients for EPS and ablation can be beneficial even if tachycardia is not induced.

We maintain that there is no group of patients in whom the probability of inducing SVT is so low that referring them for EPS would be contraindicated. If a patient has disabling symptoms then EPS is entirely reasonable. Even if SVT is not induced and no ablation is performed, the patient may take comfort in knowing that the attempt was made. There is also evidence of a placebo effect of performing EPS [6].

Predicting SVT induction and ablation at EPS is likely to remain an imperfect science. Patients who seek medical attention early in their symptomatic course will not have amassed as much objective evidence that they have a tachycardia that could be cured by ablation. Furthermore, SVT induction is not completely consistent from one attempt to the next. Induction of AVNRT, for example, is < 70% reproducible in about 10% of patients [27]. In our population there were 89 instances where patients underwent repeat EPS. In 18 of these cases (20%) the results of the 2 studies were discordant, meaning either that SVT was inducible at the second study but not the first, or that a different type of SVT was induced at the second study than at the first.

The predictive features described here allow patients and physicians to make a more informed decision about whether to pursue invasive testing, and provide a useful tool for managing patient expectations. They can offer a rationale for avoiding EPS in high‐risk patients, such as the elderly, the frail, and those with serious comorbidities. Conversely, in cases where tachycardia induction was expected but did not occur, they can reassure both the operator and patient that the procedure was indicated.

## 5 Limitations

Our study was retrospective and not blinded. We could collect data only from patients who were referred for EPS. This introduced a selection bias towards patients who were more likely to have SVT induced. In our entire population, only 12% of patients did not exhibit a criterion predicting SVT induction.

There was no standardized protocol for SVT induction. Some of the non‐inducible patients (e.g., those with pre‐excitation) may have had SVT induced if an aggressive induction protocol had been consistently followed. Most of the SVT inductions (~91%) were either spontaneous with catheter placement or were induced by atrial pacing on or off isoproterenol (Supporting Information S1: Table S2B). Most cases of non‐induction (87%) employed atrial extrastimulus testing on and off isoproterenol, and about 1/3 also employed ventricular extrastimulus testing on and off isuproterenol (Supporting Information S1: Table S2C). These data suggest that, with the possible exception of patients with pre‐excitation, attempts to elicit tachycardia in noninducible patients were sufficient.

Some of the induced tachycardias may have been spurious, that is, they may not have been the cause of the patient's symptoms. Among the 720 patients in whom SVT was both induced and ablated, a second type of SVT was induced in only 20 cases (2.8%). Assuming that only one type of SVT was clinically active, these data suggest that the incidence of spurious induction is low.

About 2/3 of the long‐term ambulatory monitors in our study (Preventice) reported a maximum episode duration of 60 s. If these monitors had reported the full duration of the longest episode, the cutoff duration best predicting SVT induction may have been longer than 30 s.

Our study did not address all factors that might affect SVT induction. We could not evaluate the effect of antiarrhythmic medications or AV nodal blocking agents because very few patients were taking them at the time of EPS and their use was poorly documented. Most importantly, we could not assess clinical judgment, which is necessarily subjective, difficult to quantify, and not consistently recorded.

## 6 Conclusion

We describe five simple preoperative criteria that predict whether patients are likely to have SVT induced and ablated at an EPS. The criteria can be incorporated into a scoring system to predict the probability of SVT induction and ablation in individual patients. They can be used to select patients for EPS and ablation of SVT, to provide a rationale for avoiding EPS in high‐risk cases, and to manage patient and operator expectations.

## Conflicts of Interest

The authors have no conflicts of interest to disclose.

## Supporting information

Supporting information.

## Data Availability

The data that support the findings of this study are available on request from the corresponding author. The data are not publicly available due to privacy or ethical restrictions.
