# Supplementary material for: Five Criteria Predict Induction and Ablation of Supraventricular Tachycardia
Source: J Cardiovasc Electrophysiol. 2024 Nov 18;36(1):220–34. doi: 10.1111/jce.16496 (PMC11727007; doi:10.1111/jce.16496)
Supplement: Supplementary file 1 — Supporting information. [file JCE-36-220-s001.docx]

**Supplementary Table S1. Patient Baseline Characteristics**

| **Feature** | **SVT Induced**  **(n=401)** | **SVT Not Induced**  **(n=186)** | **p value** |
| --- | --- | --- | --- |
| Average Age | 52.6 | 49.1 | 0.01 |
| % Male | 53% | 52% | 0.77 |
| Mean LVEF | 61% | 61% | 0.81 |
| h/o Coronary Artery Disease | 8.0% | 5.9% | 0.37 |
| h/o Cardiomyopathy or CHF | 4.5% | 7.5% | 0.13 |
| Previous Cardiac Surgery | 5.7% | 7.5% | 0.41 |
| h/o Hypertension | 30% | 34% | 0.30 |
| h/o Diabetes | 13% | 10% | 0.29 |
| h/o Hyperlipidemia | 18% | 13% | 0.13 |
| h/o Obstructive Sleep Apnea | 16% | 12% | 0.21 |
| h/o Atrial Fibrillation/Flutter | 9% | 12% | 0.38 |

**Supplementary Table S2A. Frequency of SVT Induction Among Patients Undergoing Ablation**

| **Type of Ablation** | **Total** | **SVT Induced** | **% SVT Induced** |
| --- | --- | --- | --- |
| Slow Pathway (SP) | 428 | 373 | 87% |
| Concealed Accessory Pathway (AP)* | 57 | 53 | 93% |
| Focal Atrial Tachycardia (FAT) | 70 | 59 | 84% |

*Excludes patients with manifest pre-excitation, many of whom had never experienced tachycardia and were undergoing empiric AP ablation.

**Supplementary Table S2B. Induction Maneuvers Provoking SVT in Inducible Patients**

|  | Off Isoproterenol | On Isoproterenol |
| --- | --- | --- |
| Spontaneous | 14% | 3% |
| A pacing | 32% | 42% |
| V Pacing | 6% | 3% |
| Total | 52% | 48% |

Analysis of 343 patients for whom data was available.

**Supplementary Table S2C. Induction Maneuvers Attempted in Non-Inducible Patients**

|  | Off Isoproterenol | On Isoproterenol |
| --- | --- | --- |
| A pacing | 100% | 88% |
| V Pacing | 99% | 37% |

Analysis of 136 patients for whom data was available.

**Supplementary Table S3:**

**Factors Predicting SVT Induction, Excluding Cases with Positive Factor**

| **Variable** | **Variable Present** | | **Variable Absent** | | **RR** | **P value** |
| --- | --- | --- | --- | --- | --- | --- |
|  | **Inducible / Total** | **Inducible**  **%** | **Inducible / Total** | **Inducible**  **%** |  |  |
| **A. EKG Features** | | | | | | |
| EKG with Irregular SVT | 2 / 12 | 17 | 15 / 78 | 19 | 0.87 | 0.84 |
| EKG showing Start or Stop | 2 / 11 | 18 | 15 / 79 | 19 | 0.96 | 0.95 |
| EKG c/w ST, AF, or AFL | 0 / 9 | 0 | 17 / 81 | 21 | 0.00 | <0.001 |
| Non-typical SVT EKG | 2 / 20 | 10 | 15 / 70 | 21 | 0.47 | 0.29 |
| **B. Response to Adenosine or Vagal Maneuvers** | | | | | | |
| Adenosine Failure | 1 / 8 | 13 | 16 / 82 | 20 | 0.64 | 0.65 |
| Vagal Failure | 1 / 7 | 14 | 16 / 83 | 19 | 0.74 | 0.75 |
| Adenosine or Vagal Failure | 2 / 13 | 15 | 15 / 77 | 19 | 0.79 | 0.73 |
| **C. Cardiac Monitor Features** | | | | | | |
| Monitor Events vs No Events | 7 / 33 | 21 | 6 / 33 | 18 | 1.17 | 0.76 |
| Longest Event Regular vs No Events | 4 / 10 | 40 | 6 / 33 | 18 | 2.20 | 0.15 |
| Longest Event Irregular vs No Events | 1 / 12 | 8 | 6 / 33 | 18 | 0.46 | 0.45 |
| Longest Event No P Wave vs No Events | 3 / 9 | 33 | 6 / 33 | 18 | 1.83 | 0.32 |
| Longest Event Short RP Pattern vs No Events | 1 / 2 | 50 | 6 / 33 | 18 | 2.75 | 0.21 |
| Longest Event Long RP Pattern vs No Events | 1 / 12 | 8 | 6 / 33 | 18 | 0.46 | 0.45 |
| Symptoms Correlated with SVT vs No Events | 2 / 9 | 22 | 6 / 33 | 18 | 1.22 | 0.78 |
| **D. Clinical Features** | | | | | | |
| Male Sex vs Female Sex | 5 / 39 | 13 | 12 / 51 | 24 | 0.54 | 0.22 |
| Incidental Presentation | 0 / 3 | 0 | 17 / 87 | 20 | 0.00 | <0.001 |
| Previous EPS for SVT | 2 / 12 | 17 | 15 / 78 | 19 | 0.87 | 0.84 |
| EPS at University vs VA | 16 / 71 | 23 | 1 / 19 | 5 | 4.28 | 0.15 |

**Supplementary Table S4: Factors Predicting SVT Ablation, Derivation Cohort**

| **Variable** | **Variable Present** | | **Variable Absent** | | **RR** | **P value** |
| --- | --- | --- | --- | --- | --- | --- |
|  | **Ablated / Total** | **%**  **Ablated** | **Ablated / Total** | **%**  **Ablated** |  |  |
| **A. EKG Features** | | | | | | |
| SVT Captured on EKG | 329 / 372 | 88 | 314 / 436 | 72 | 1.23 | <0.001 |
| EKG with Regular SVT | 276 / 300 | 92 | 367 / 508 | 72 | 1.27 | <0.001 |
| EKG with Irregular SVT | 11 / 22 | 50 | 632 / 786 | 80 | 0.62 | 0.026 |
| EKG showing Start or Stop | 11 / 22 | 50 | 632 / 786 | 80 | 0.62 | 0.026 |
| EKG c/w ST, AF, or AFL | 7 / 17 | 41 | 636 / 791 | 80 | 0.51 | 0.021 |
| Typical SVT EKG | 267 / 283 | 94 | 334 / 477 | 70 | 1.35 | <0.001 |
| Non-Typical SVT EKG | 20 / 41 | 49 | 581 / 719 | 81 | 0.60 | 0.002 |
| Typical EKG, No P Wave | 95 / 102 | 93 | 548 / 706 | 78 | 1.20 | <0.001 |
| Typical EKG, rSr’ Pattern | 95 / 98 | 97 | 548 / 710 | 77 | 1.26 | <0.001 |
| Typical EKG, Short RP | 62 / 67 | 93 | 581 / 741 | 78 | 1.18 | <0.001 |
| Typical EKG, Long RP | 23 / 24 | 96 | 620 / 784 | 79 | 1.21 | <0.001 |
| **B. Termination with Adenosine or Vagal Maneuvers** | | | | | | |
| Termination with Adenosine | 221 / 233 | 95 | 422 / 573 | 74 | 1.29 | <0.001 |
| Termination with Adenosine without SVT EKG | 55 / 61 | 90 | 321 / 462 | 69 | 1.30 | <0.001 |
| Failure to Terminate with Adenosine | 12 / 22 | 55 | 631 / 784 | 80 | 0.68 | 0.047 |
| Termination with Vagal Stimulus | 193 / 219 | 88 | 452 / 591 | 76 | 1.15 | <0.001 |
| Termination with Vagal Stimulus without SVT EKG | 101 / 122 | 83 | 277 / 405 | 68 | 1.21 | <0.001 |
| Failure to Terminate with Vagal Stimulus | 74 / 86 | 86 | 571 / 724 | 79 | 1.09 | 0.067 |
| Vagal Failure with Termination by Adenosine | 52 / 53 | 98 | 591 / 753 | 78 | 1.25 | <0.001 |
| Vagal Failure without Termination by Adenosine | 21 / 32 | 66 | 401 / 541 | 74 | 0.89 | 0.35 |

| **C. Cardiac Monitor Features** | | | | | | |
| --- | --- | --- | --- | --- | --- | --- |
| Monitor Events vs No Events | 250 / 320 | 78 | 97 / 141 | 69 | 1.14 | 0.047 |
| Longest Event Regular vs No Events | 200 / 227 | 88 | 97 / 141 | 69 | 1.28 | <0.001 |
| Longest Event Irregular vs No Events | 23 / 42 | 55 | 97 / 141 | 69 | 0.80 | 0.13 |
| Longest Event ≥30 sec vs <30 sec | 228 / 268 | 85 | 124/206 | 60 | 1.41 | <0.001 |
| Longest Event ≥30 sec without SVT EKG or Adenosine/Vagal Termination | 124 / 157 | 79 | 45 / 107 | 42 | 1.88 | <0.001 |
| Symptoms Correlated with SVT vs No Events | 166 / 192 | 86 | 97 / 141 | 69 | 1.26 | <0.001 |
| Symptoms Correlated with SVT vs No Events, Excluding Longest Event ≥30 sec | 23 / 34 | 68 | 97 / 141 | 69 | 0.98 | 0.90 |
| Symptoms not Correlated with SVT vs No Events | 72 / 105 | 69 | 97 / 141 | 69 | 1.00 | 0.97 |
| Longest Event No P Wave vs No Events | 164 / 184 | 89 | 97 / 141 | 69 | 1.30 | <0.001 |
| Longest Event rSr' Pattern vs No Events | 7 / 9 | 78 | 97 / 141 | 69 | 1.13 | 0.51 |
| Longest Event Short RP Pattern vs No Events | 19 / 21 | 90 | 97 / 141 | 69 | 1.32 | 0.003 |
| Longest Event Long RP Pattern vs No Events | 32 / 55 | 58 | 97 / 141 | 69 | 0.85 | 0.19 |
| **D. Pre-Excitation** | | | | | | |
| All Pre-excited Patients | 72 / 79 | 91 | 558 / 713 | 78 | 1.16 | <0.001 |
| Asymptomatic Pre-excitation | 24 / 25 | 96 | 606 / 767 | 79 | 1.22 | <0.001 |
| Symptomatic Pre-Excitation | 48 / 54 | 89 | 582 / 738 | 79 | 1.13 | 0.021 |
| All Pre-excited Patients Excl. SVT EKG, Adenosine/Vagal Termination, or Event ≥30 sec | 46 / 51 | 90 | 20 / 84 | 24 | 3.79 | <0.001 |

| **E. Clinical Features** | | | | | | |
| --- | --- | --- | --- | --- | --- | --- |
| Age ≥ 50 | 369 / 454 | 81 | 276 / 356 | 78 | 1.05 | 0.19 |
| Male Sex vs Female Sex | 351 / 428 | 82 | 294 / 382 | 77 | 1.07 | 0.078 |
| Previous EPS for SVT | 56 / 73 | 77 | 589 / 737 | 80 | 0.96 | 0.54 |
| History of Syncope | 82 / 120 | 68 | 563 / 690 | 82 | 0.84 | 0.006 |
| History of Syncope  Excluding (+) Factor | 5 / 21 | 24 | 18 / 69 | 26 | 0.91 | 0.84 |
| Sudden onset vs Not Sudden | 214 / 255 | 84 | 69 / 100 | 69 | 1.22 | 0.007 |
| Sudden onset vs Not Sudden Excluding (+) Factor | 6 / 20 | 30 | 5 / 16 | 31 | 0.96 | 0.94 |
| ER Presentation | 360 / 420 | 86 | 285 / 390 | 73 | 1.17 | <0.001 |
| ER Presentation  Excluding (+) Factor | 8 / 26 | 31 | 15 / 64 | 23 | 1.31 | 0.47 |
| Clinic Presentation | 209 / 298 | 70 | 436 / 512 | 85 | 0.82 | <0.001 |
| Clinic Presentation  Excluding (+) Factor | 14 / 59 | 24 | 9 / 31 | 29 | 0.82 | 0.58 |
| Incidental Presentation | 70 / 84 | 83 | 575 / 726 | 79 | 1.05 | 0.33 |
| EPS at University vs VA | 478 / 602 | 79 | 167 / 208 | 80 | 0.99 | 0.78 |

**Supplementary** **Table S5. Independence of Features Predicting SVT Induction and Ablation**

| **SVT Induction** | | | | | | |
| --- | --- | --- | --- | --- | --- | --- |
| **Variable** | **Variable Present** | | **Variable Absent** | | **RR** | **P value** |
|  | **Induced / Total** | **%**  **Induced** | **Induced / Total** | **%**  **Induced** |  |  |
| Termination with Adenosine | 24 / 30 | 80 | 15 / 84 | 18 | 4.48 | <0.001 |
| Typical SVT EKG | 46 / 58 | 79 | 15 / 84 | 18 | 4.44 | <0.001 |
| Monitor Event ≥30 sec | 94 / 149 | 63 | 15 / 84 | 18 | 3.53 | <0.001 |
| Term with Vagal Maneuvers | 23 / 39 | 59 | 15 / 84 | 18 | 3.30 | <0.001 |
| Pre-Excitation | 21 / 51 | 41 | 15 / 84 | 18 | 2.31 | 0.004 |
| **SVT Ablation** | | | | | | |
| **Variable** | **Variable Present** | | **Variable Absent** | | **RR** | **P value** |
|  | **Ablated / Total** | **%**  **Ablated** | **Ablated / Total** | **%**  **Ablated** |  |  |
| Termination with Adenosine | 27 / 30 | 90 | 20 / 84 | 24 | 3.78 | <0.001 |
| Typical SVT EKG | 50 / 58 | 86 | 20 / 84 | 24 | 3.62 | <0.001 |
| Monitor Event ≥30 sec | 117 / 149 | 79 | 20 / 84 | 24 | 3.30 | <0.001 |
| Term with Vagal Maneuvers | 24 / 39 | 62 | 20 / 84 | 24 | 2.58 | <0.001 |
| Pre-Excitation | 46 / 51 | 90 | 20 / 84 | 24 | 3.79 | <0.001 |

**Supplementary Table S6: Factors Predicting SVT Induction, Validation Cohort**

| **Variable** | **Variable Present** | | **Variable Absent** | | **RR** | **P value** |
| --- | --- | --- | --- | --- | --- | --- |
|  | **Inducible / Total** | **Inducible**  **%** | **Inducible / Total** | **Inducible**  **%** |  |  |
| **A. EKG Features** | | | | | | |
| SVT Captured on EKG | 106 / 127 | 83 | 85 / 141 | 60 | 1.38 | <0.001 |
| EKG with Regular SVT | 96 / 112 | 86 | 95 / 156 | 61 | 1.41 | <0.001 |
| EKG with Irregular SVT | 3 / 7 | 43 | 188 / 261 | 72 | 0.59 | 0.24 |
| EKG showing Start or Stop | 3 / 8 | 38 | 188 / 260 | 72 | 0.52 | 0.15 |
| EKG c/w ST, AF, or AFL | 2 / 3 | 67 | 189 / 265 | 71 | 0.93 | 0.87 |
| Typical SVT EKG | 93 / 106 | 88 | 90 / 153 | 59 | 1.49 | <0.001 |
| Non-Typical SVT EKG | 5 / 12 | 42 | 178 / 247 | 72 | 0.58 | 0.11 |
| Typical EKG, No P Wave | 36 / 43 | 84 | 155 / 225 | 69 | 1.22 | 0.016 |
| Typical EKG, rSr’ Pattern | 27 / 31 | 87 | 164 / 237 | 69 | 1.26 | 0.005 |
| Typical EKG, Short RP | 18 / 19 | 95 | 173 / 249 | 69 | 1.36 | <0.001 |
| Typical EKG, Long RP | 11 / 12 | 92 | 180 / 256 | 70 | 1.30 | 0.006 |
| **B. Termination with Adenosine or Vagal Maneuvers** | | | | | | |
| Termination with Adenosine | 68 / 77 | 88 | 131 / 202 | 65 | 1.36 | <0.001 |
| Termination with Adenosine without SVT EKG | 16 / 16 | 100 | 90 / 157 | 57 | 1.74 | <0.001 |
| Failure to Terminate with Adenosine | 9 / 12 | 75 | 190/267 | 71 | 1.05 | 0.76 |
| Termination with Vagal Stimulus | 66 / 73 | 90 | 133 / 206 | 65 | 1.40 | <0.001 |
| Termination with Vagal Stimulus without SVT EKG | 37 / 41 | 90 | 69 / 132 | 52 | 1.73 | <0.001 |
| Failure to Terminate with Vagal Stimulus | 26 / 31 | 84 | 173 / 248 | 70 | 1.20 | 0.039 |
| Vagal Failure with Termination by Adenosine | 20 / 24 | 83 | 179 / 255 | 70 | 1.19 | 0.087 |
| Vagal Failure without Termination by Adenosine | 6 / 7 | 86 | 125 / 195 | 64 | 1.34 | 0.076 |

| **C. Cardiac Monitor Features** | | | | | | |
| --- | --- | --- | --- | --- | --- | --- |
| Monitor Events vs No Events | 108 / 146 | 74 | 40 / 70 | 57 | 1.29 | 0.025 |
| Longest Event Regular vs No Events | 90 / 113 | 80 | 40 / 70 | 57 | 1.39 | 0.004 |
| Longest Event Irregular vs No Events | 7 / 17 | 41 | 40 / 70 | 57 | 0.72 | 0.29 |
| Longest Event ≥30 sec vs <30 sec | 93 / 109 | 85 | 55 / 108 | 51 | 1.68 | <0.001 |
| Longest Event ≥30 sec without SVT EKG or Adenosine/Vagal Termination | 38 / 52 | 73 | 11 / 55 | 20 | 3.65 | <0.001 |
| Symptoms Correlated with SVT vs No Events | 72 / 89 | 81 | 40 / 70 | 57 | 1.42 | 0.003 |
| Symptoms Correlated with SVT vs No Events, Excluding Longest Event ≥30 sec | 9 / 18 | 50 | 40 / 70 | 57 | 0.88 | 0.61 |
| Symptoms not Correlated with SVT vs No Events | 27 / 43 | 63 | 40 / 70 | 57 | 1.10 | 0.55 |
| Longest Event No P Wave vs No Events | 66 / 82 | 80 | 40 / 70 | 57 | 1.41 | 0.003 |
| Longest Event rSr' Pattern vs No Events | 2 / 3 | 67 | 40 / 70 | 57 | 1.17 | 0.72 |
| Longest Event Short RP Pattern vs No Events | 10 / 13 | 77 | 40 / 70 | 57 | 1.35 | 0.11 |
| Longest Event Long RP Pattern vs No Events | 14 / 27 | 52 | 40 / 70 | 57 | 0.91 | 0.65 |
| **D. Pre-Excitation** | | | | | | |
| All Pre-excited Patients | 21 / 41 | 51 | 172 / 229 | 75 | 0.68 | 0.015 |
| Asymptomatic Pre-excitation | 3 / 14 | 21 | 190 / 256 | 74 | 0.29 | 0.016 |
| Symptomatic Pre-Excitation | 18 / 25 | 72 | 175 / 245 | 71 | 1.01 | 0.95 |
| All Pre-excited Patients Excl. SVT EKG, Adenosine/Vagal Termination, or Event ≥30 sec | 6 / 23 | 26 | 7 / 33 | 21 | 1.23 | 0.67 |
| Asymptomatic Pre-excitation, Excluding those with (+) Feature | 2 / 11 | 18 | 11 / 45 | 24 | 0.74 | 0.67 |
| Symptomatic Pre-excitation, Excluding those with (+) Feature | 4 / 11 | 36 | 9 / 45 | 20 | 1.82 | 0.23 |

| **E. Clinical Features** | | | | | | |
| --- | --- | --- | --- | --- | --- | --- |
| Age ≥ 50 | 115 / 146 | 79 | 84 / 133 | 63 | 1.25 | 0.005 |
| Age ≥ 50  Excluding (+) Factor | 2 / 15 | 13 | 11 / 43 | 26 | 0.52 | 0.36 |
| Male Sex vs Female Sex | 95 / 140 | 68 | 103 / 138 | 75 | 0.91 | 0.21 |
| Previous EPS for SVT | 22 / 30 | 73 | 177 / 249 | 71 | 1.03 | 0.79 |
| History of Syncope | 19 / 36 | 53 | 180 / 243 | 74 | 0.71 | 0.037 |
| History of Syncope  Excluding (+) Factor | 0 / 5 | 0 | 7 / 30 | 23 | 0.00 | <0.001 |
| Sudden onset vs Not Sudden | 56 / 74 | 76 | 3 / 3 | 100 | 0.76 | <0.001 |
| Sudden onset vs Not Sudden Excluding (+) Factor | 4 / 8 | 50 | 0 / 0 | ND | ND | ND |
| ER Presentation | 121 / 149 | 81 | 78 / 130 | 60 | 1.35 | <0.001 |
| ER Presentation  Excluding (+) Factor | 4 / 10 | 40 | 3 / 25 | 12 | 3.33 | 0.075 |
| Clinic Presentation | 63 / 98 | 64 | 136 / 181 | 75 | 0.86 | 0.072 |
| Clinic Presentation  Excluding (+) Factor | 3 / 23 | 13 | 4 / 12 | 33 | 0.39 | 0.17 |
| Incidental Presentation | 14 / 29 | 48 | 185 / 250 | 74 | 0.65 | 0.029 |
| EPS at University vs VA | 170 / 242 | 70 | 27 / 34 | 79 | 0.88 | 0.21 |

**Supplementary Table S7: Factors Predicting SVT Ablation, Validation Cohort**

| **Variable** | **Variable Present** | | **Variable Absent** | | **RR** | **P value** |
| --- | --- | --- | --- | --- | --- | --- |
|  | **Inducible / Total** | **Inducible**  **%** | **Inducible / Total** | **Inducible**  **%** |  |  |
| **A. EKG Features** | | | | | | |
| SVT Captured on EKG | 110 / 127 | 87 | 102 / 141 | 72 | 1.20 | 0.004 |
| EKG with Regular SVT | 98 / 112 | 88 | 114 / 156 | 73 | 1.20 | 0.003 |
| EKG with Irregular SVT | 5 / 7 | 71 | 207 / 261 | 79 | 0.90 | 0.67 |
| EKG showing Start or Stop | 5 / 8 | 63 | 207 / 260 | 80 | 0.79 | 0.38 |
| EKG c/w ST, AF, or AFL | 2 / 3 | 67 | 210 / 265 | 79 | 0.84 | 0.67 |
| Typical SVT EKG | 95 / 106 | 90 | 109 / 153 | 71 | 1.26 | <0.001 |
| Non-Typical SVT EKG | 7 / 12 | 58 | 197 / 247 | 80 | 0.73 | 0.21 |
| Typical EKG, No P Wave | 39 / 43 | 91 | 173 / 225 | 77 | 1.18 | 0.007 |
| Typical EKG, rSr’ Pattern | 28 / 31 | 90 | 184 / 237 | 78 | 1.16 | 0.027 |
| Typical EKG, Short RP | 18 / 19 | 95 | 194 / 249 | 78 | 1.22 | 0.002 |
| Typical EKG, Long RP | 9 / 12 | 75 | 203 / 256 | 79 | 0.95 | 0.74 |
| **B. Termination with Adenosine or Vagal Maneuvers** | | | | | | |
| Termination with Adenosine | 72 / 77 | 94 | 149 / 202 | 74 | 1.27 | <0.001 |
| Termination with Adenosine without SVT EKG | 16 / 16 | 100 | 110 / 157 | 70 | 1.43 | <0.001 |
| Failure to Terminate with Adenosine | 10 / 12 | 83 | 211 / 267 | 79 | 1.05 | 0.69 |
| Termination with Vagal Stimulus | 68 / 73 | 93 | 153 / 206 | 74 | 1.25 | <0.001 |
| Termination with Vagal Stimulus without SVT EKG | 37 / 41 | 90 | 89 / 132 | 67 | 1.34 | <0.001 |
| Failure to Terminate with Vagal Stimulus | 28 / 31 | 90 | 193 / 248 | 78 | 1.16 | 0.028 |
| Vagal Failure with Termination by Adenosine | 22 / 24 | 92 | 199 / 255 | 78 | 1.17 | 0.022 |
| Vagal Failure without Termination by Adenosine | 6 / 7 | 86 | 143 / 195 | 73 | 1.17 | 0.33 |
| **C. Cardiac Monitor Features** | | | | | | |
| Monitor Events vs No Events | 115 / 146 | 79 | 49 / 70 | 70 | 1.13 | 0.19 |
| Longest Event Regular vs No Events | 93 / 113 | 82 | 49 / 70 | 70 | 1.18 | 0.072 |
| Longest Event Irregular vs No Events | 10 / 17 | 59 | 49 / 70 | 70 | 0.84 | 0.43 |
| Longest Event ≥30 sec vs <30 sec | 91 / 109 | 83 | 72 / 108 | 67 | 1.25 | 0.005 |
| Longest Event ≥30 sec without SVT EKG or Adenosine/Vagal Termination | 41 / 52 | 79 | 25 / 55 | 45 | 1.73 | 0.001 |
| Symptoms Correlated with SVT vs No Events | 71 / 89 | 80 | 49 / 70 | 70 | 1.14 | 0.17 |
| Symptoms Correlated with SVT vs No Events, Excluding Longest Event ≥30 sec | 10 / 18 | 56 | 49 / 70 | 70 | 0.79 | 0.31 |
| Symptoms not Correlated with SVT vs No Events | 34 / 43 | 79 | 49 / 70 | 70 | 1.13 | 0.27 |
| Longest Event No P Wave vs No Events | 71 / 82 | 87 | 49 / 70 | 70 | 1.24 | 0.018 |
| Longest Event rSr' Pattern vs No Events | 1 / 3 | 33 | 49 / 70 | 70 | 0.48 | 0.37 |
| Longest Event Short RP Pattern vs No Events | 9 / 13 | 69 | 49 / 70 | 70 | 0.99 | 0.96 |
| Longest Event Long RP Pattern vs No Events | 17 / 27 | 63 | 49 / 70 | 70 | 0.90 | 0.53 |
| **D. Pre-Excitation** | | | | | | |
| All Pre-excited Patients | 38 / 41 | 93 | 176 / 229 | 77 | 1.21 | 0.001 |
| Asymptomatic Pre-excitation | 13 / 14 | 93 | 201 / 256 | 79 | 1.18 | 0.039 |
| Symptomatic Pre-Excitation | 24 / 25 | 96 | 190 / 245 | 78 | 1.24 | <0.001 |
| All Pre-excited Patients Excl. SVT EKG, Adenosine/Vagal Termination, or Event ≥30 sec | 20 / 23 | 87 | 10 / 33 | 30 | 2.87 | <0.001 |
| Asymptomatic Pre-excitation, Excluding those with (+) Feature | 10 /11 | 91 | 20 / 45 | 44 | 2.05 | <0.001 |
| Symptomatic Pre-excitation, Excluding those with (+) Feature | 10 /11 | 91 | 20 / 45 | 44 | 2.05 | <0.001 |
| **E. Clinical Features** | | | | | | |
| Age ≥ 50 | 123 / 146 | 84 | 98 / 133 | 74 | 1.14 | 0.034 |
| Age ≥ 50  Excluding (+) Factor | 8 / 15 | 53 | 22 / 43 | 51 | 1.04 | 0.89 |
| Male Sex vs Female Sex | 111 / 140 | 79 | 109 / 138 | 79 | 1.00 | 0.95 |
| Previous EPS for SVT | 24 / 30 | 80 | 197 / 249 | 79 | 1.01 | 0.91 |
| History of Syncope | 26 / 36 | 72 | 195 / 243 | 80 | 0.90 | 0.33 |
| History of Syncope  Excluding (+) Factor | 0 / 5 | 0 | 10 / 30 | 33 | 0.00 | <0.001 |
| Sudden onset vs Not Sudden | 60 / 74 | 81 | 3 / 3 | 100 | 0.81 | <0.001 |
| Sudden onset vs Not Sudden Excluding (+) Factor | 4 / 8 | 50 | 0 / 0 | ND | ND | ND |
| ER Presentation | 127 / 149 | 85 | 94 / 130 | 72 | 1.18 | 0.01 |
| ER Presentation  Excluding (+) Factor | 5 / 10 | 50 | 5 / 25 | 20 | 2.50 | 0.077 |
| Clinic Presentation | 69 /98 | 70 | 152 / 181 | 84 | 0.84 | 0.016 |
| Clinic Presentation  Excluding (+) Factor | 5 / 23 | 22 | 5 / 12 | 42 | 0.52 | 0.22 |
| Incidental Presentation | 23 / 29 | 79 | 198 / 250 | 79 | 1.00 | 0.99 |
| EPS at University vs VA | 191 / 242 | 79 | 28 / 34 | 82 | 0.96 | 0.62 |
